# Do-not-resuscitate orders in patients with community-acquired pneumonia: a retrospective study

**DOI:** 10.1186/s12890-020-01236-1

**Published:** 2020-07-24

**Authors:** Gertrud Baunbæk Egelund, Andreas Vestergaard Jensen, Pelle Trier Petersen, Stine Bang Andersen, Bjarne Ørskov Lindhardt, Gernot Rohde, Pernille Ravn, Christian von Plessen

**Affiliations:** 1 Department of Pulmonary and infectious medicine, Nordsjællands Hospital, Dyrehavevej 29, 3400 Hillerød, Denmark; 2 University of Copenhagen, Faculty of Health and Medical Sciences, Copenhagen, Denmark; 3 CAPNETZ-Stiftung, Hannover Medical School, Hanover, Germany; 4 Department of Infectious Diseases, Amager Hvidovre Hospital, Hvidovre, Denmark; 5 Department of Respiratory Medicine, Medical Clinic I, Goethe University Hospital, Frankfurt, Germany; 6 Department of Medicine, Unit for Infectious Diseases, Herlev Gentofte Hospital, Hellerup, Denmark; 7 Institute for Clinical research University of Southern Denmark, Campusvej 55, DK-5230 Odense M, Denmark; 8 Unisanté Rue du Bugnon 44, CH-1011, Lausanne, Switzerland

**Keywords:** Community-acquired pneumonia, Do not resuscitate orders, Mortality

## Abstract

**Background:**

To investigate the use of do-not-resuscitate (DNR) orders in patients hospitalized with community-acquired pneumonia (CAP) and the association with mortality.

**Methods:**

We assembled a cohort of 1317 adults hospitalized with radiographically confirmed CAP in three Danish hospitals. Patients were grouped into no DNR order, early DNR order (≤48 h after admission), and late DNR order (> 48 h after admission). We tested for associations between a DNR order and mortality using a cox proportional hazard model adjusted for patient and disease related factors.

**Results:**

Among 1317 patients 177 (13%) patients received a DNR order: 107 (8%) early and 70 (5%) late, during admission. Patients with a DNR order were older (82 years vs. 70 years, *p* < 0.001), more frequently nursing home residents (41% vs. 6%, p < 0.001) and had more comorbidities (one or more comorbidities: 73% vs. 59%, p < 0.001). The 30-day mortality was 62% and 4% in patients with and without a DNR order, respectively. DNR orders were associated with increased risk of 30-day mortality after adjustment for age, nursing home residency and comorbidities. The association was modified by the CURB-65 score Hazard ratio (HR) 39.3 (95% CI 13.9–110.6), HR 24.0 (95% CI 11.9–48,3) and HR 9.4 (95% CI: 4.7–18.6) for CURB-65 score 0–1, 2 and 3–5, respectively.

**Conclusion:**

In this representative Danish cohort, 13% of patients hospitalized with CAP received a DNR order. DNR orders were associated with higher mortality after adjustment for clinical risk factors. Thus, we encourage researcher to take DNR orders into account as potential confounder when reporting CAP associated mortality.

## Background

Do-not-resuscitate (DNR) orders are among, the most difficult clinical decisions. On the one hand, it is clearly not always in a patient’s best interest to try to prolong a life with severe chronic disease and suffering. On the other hand, a DNR order documents a decision to withhold a potentially life-saving treatment and has been associated with excess mortality [1, 2]. A better understanding of the associations between DNR orders and mortality could aid clinicians in these difficult decisions.

Community-acquired pneumonia (CAP) is one of the most common infectious diseases in the western world, especially in elderly patients [3–5]. All physicians, from primary to intensive care, will treat patients with CAP [6]. Mortality has been persistently high during the past decades and CAP is still a leading cause of mortality worldwide [4, 7–11]. Older age, comorbidities and frailty contribute to the high mortality [12–15]. Given the high incidence, mortality and complicated mix of prognostic factors in patients with CAP, it is surprising that only few studies [16–19] have investigated the influence of DNR orders on clinical outcomes. In fact, previous studies have indicated that a DNR order is associated with mortality [19].

Therefore, when assessing the mortality for epidemiological or quality improvement purposes, it is possible that DNR orders should be considered as a potential independent risk factor for excess mortality in the large group of patients with CAP. Thus, in this study, we investigate the use of DNR orders in patients hospitalized with CAP and the association between DNR orders and mortality.

## Methods

### DNR orders in Denmark

In Denmark, the treating physician can write a DNR order if a patient is inevitably dying, severely disabled or in a vegetative state. DNR orders are also justified when a treatment might lead to survival with disease related consequences that are considered too severe or likely to cause suffering. The decision for a DNR order is primarily made by the treating physician. A patient cannot demand a certain treatment, including resuscitation. However, the patient can always refuse treatment, including resuscitation. This latter would also lead to a DNR order. Patients must be informed that a decision not to attempt resuscitation has been made. A DNR order is only valid for the current hospital admission and must be re-assessed upon a new admission unless the patient has written a living will refusing resuscitation [20].

### Study population and data collection

We retrospectively included immunocompetent adult patients with CAP admitted to one of three hospitals; one regional and two local hospitals in North Zealand, Denmark from January 1st 2011 until June 30th 2012.

CAP was defined as a new infiltrate on the chest X-ray and at least one of the following symptoms of lower respiratory tract infection: cough, purulent expectoration, fever or pathological lung auscultation. Exclusion criteria were hospital admission within the last 28 days, active tuberculosis or immunosuppression. The in- and exclusion criteria are described in detail in [3].

All data stem from the electronic patient records as well as laboratory, microbiological and radiological databases. We registered data into the CAPNETZ database (www.capnetz.de) and locally into EpiData entry 3.1 (www.epidata.com). Details of this CAP-North cohort are described in [3].

### Outcome measures, exposures and confounders

Our primary outcome was 30-day mortality. We grouped patients into no DNR order or DNR order. No DNR order was assumed when *“full resuscitation”* was stated in the patient record or resuscitation was not mentioned. We further grouped patients with a DNR order into early DNR (registered within 48 h of admission) or a late DNR order (registered 48 h or more after admission). We viewed age, nursing home residency, number of comorbidities (none, one or more than one), the CURB-65 score and admission to the intensive care unit (ICU) as potential confounders. The comorbidities registered in this study was COPD, other chronic respiratory disease, heart failure, other chronic heart disease, chronic kidney disease, chronic liver disease, neurological chronic disease, diabetes and malignancy.

The CURB-65 score was used as a grouped variable with three levels; CURB-65 score 0–1, CURB-65 score 2 and CURB-65 score 3–5, corresponding to mild, moderate and severe pneumonia. In Denmark, people are mainly living at a nursing home if they are not able to take care of them self either due to old age, physical or mental health issues.

### Statistical methods

Categorical variables were presented as percentages (counts) and continuous variables as medians (interquartile range (IQR)). Groups were compared with the Wilcoxon rank sum, the chi-square or the Fisher exact test. In comparisons of more than one group, we used the Kruskal-Wallis and the Chi-square test. *P*-values were adjusted for multiple comparisons using the Bonferroni correction.

We assessed the association between DNR orders and mortality with a Kaplan Meier curve and the log rank test and applied an adjusted cox proportional hazard model. The assumption of proportional hazard was assessed graphically and by assessing the martingale residuals. DNR order was included as a time dependent variable to overcome the risk of immortal time bias. Interaction analyses were performed to assess whether the effect of a DNR order was modified by other prognostic factors.

In patients with no DNR order, we tested for risk factors associated with 30-day mortality with the cox proportional hazard model. Variables significantly associated (*P* < 0.1) with 30-day mortality in the crude analysis were included in the adjusted model.

We used SAS Enterprise Guide version 7.1 and GraphPad Prism 7.02.197 R2.

## Results

Of the 1320 patients in the CAP-North cohort, DNR status could be assessed in 1317 patients; three patients were not included due to missing records. 13% (177) had a DNR order; 8% (107) had an early and 5% (70) a late DNR order. Patients with a DNR order were older (82 vs. 70 years, *p* < 0.001), more frequently nursing home residents (41% vs. 6%, p < 0.001) and had more comorbidities (73% vs. 59%, p < 0.001). Patients with a DNR order were also more severely ill (CURB-65 score ≥ 3; 50% vs 13%, *p* < 0.001) and were more likely to be admitted to the ICU (27% vs 7%, p < 0.001), Table 1. All patients, regardless of DNR-status, received antibiotics upon admission. More patients with a DNR order received combination therapy (24% vs. 14%, *p* = 0.001) and fewer small spectrum penicillin (31% vs 47%, *p* < 0.001).

**Table 1:** Baseline characteristics according to do not resuscitate status

	No DNR order *n* = 1140	DNR order *n* = 177	*P*-value
**Age**, median (IQR)	70 (55–80)	82 (73–88)	< 0.001
**Sex**, male	47% (539)	48% (85)	0.85
**Nursing home**	6% (73)	41% (72)	< 0.001
**Comorbidities**			
COPD	18% (200)	24% (42)	0.045
Oher chronic pulmonary disease	13% (143)	6% (11)	0.014
Malignancy	9% (101)	10% (18)	0.57
Chronic heart disease	20% (223)	32% (57)	< 0.001
Chronic neurological disease	12% (132)	29% (51)	< 0.001
Diabetes	13% (144)	10% (17)	0.25
Chronic liver disease	1% (9)	2% (3)	0.24
Chronic kidney disease	3% (37)	2% (4)	0.48
**Number of comorbidities**			
None	41% (468)	27% (48)	0.001
One	35% (395)	41% (71)	0.45
More than one	24% (267)	32% (56)	0.05
**Severity**			
CURB-65 0–1	59% (582)	15% (22)	< 0.001
CURB-65 2	28% (281)	34% (49)	0.43
CURB-65 3–5	13% (129)	50% (72)	< 0.001
ICU	7% (79)	27% (47)	< 0.001
Intubation	3% (35)	12% (22)	< 0.001
**Antibiotic treatment**^a^			
Monotherapy penicillin^b^	47% (535)	31% (54)	< 0.001
Monotherapy other beta-lactam	29% (332)	36% (63)	0.08
Combination therapy	14% (163)	24% (43)	0.001
Other	10% (110)	10% (17)	0.99
**Mortality**			
30-day	4% (40)	62% (109)	< 0.001
90-day	6% (69)	75% (133)	< 0.001
180-day	9% (102)	81% (143)	< 0.001

^a^Empiric therapy. ^b^ benzylpenicillin or phenoxymethyl penicillin. COPD: chronic obstructive pulmonary disease. ICU: intensive care unit. IQR: interquartile range

Patients with an early DNR order were older (85 vs. 79, *p* = 0.002) and more of them were nursing home residents (53 vs. 23, p < 0.001) compared with patients with a late DNR order. Further, patients with an early DNR order were more severely ill upon admission (CURB-65 score ≥ 3: 59% vs. 38%, *p* = 0.04), but less likely to be admitted to the ICU (20% vs 36%, *p* = 0.03) compared with patients with a late DNR order. The number of comorbidities (one or more comorbidities; 75% vs. 69%, *p* = 0.78) was not significantly different between patients with early and late DNR orders (Table 2).

**Table 2:** Baseline characteristics according to early or late do not resuscitate order

	Early DNR order *n* = 107	Late DNR order *n* = 70	***p***-value
**Age**, median (IQR)	85 (76–89)	79 (72–85)	0.001
**Sex**, male	46% (49)	51% (36)	0.46
**Nursing home**	53% (56)	23% (16)	< 0.001
**Comorbidities**			
COPD	22% (23)	27% (19)	0.43
Oher chronic pulmonary disease	6% (6)	7% (5)	0.68
Malignancy	11% (12)	9% (6)	0.57
Chronic heart disease	38% (41)	23% (16)	0.03
Chronic neurological disease	33% (35)	23% (16)	0.16
Diabetes	7% (8)	13% (9)	0.24
Chronic liver disease	0	4% (3)	0.03
Chronic kidney disease	3% (3)	1% (1)	0.55
**Number of comorbidities**			
None	25% (26)	31% (22)	0.99
One	40% (42)	41% (29)	1.00
More than one	35% (37)	27% (19)	0.78
**Severity**			
CURB-65 0–1	11% (9)	22% (13)	0.46
CURB-65 2	31% (26)	40% (23)	0.78
CURB-65 3–5	59% (50)	38% (22)	0.04
ICU	20% (22)	36% (25)	0.03
Intubation	4% (4)	26% (17)	< 0.001
**Antibiotic treatment**^a^			
Monotherapy penicillin^b^	31% (33)	30% (21)	0.91
Monotherapy other betalactam	33% (35)	40% (28)	0.32
Combination therapy	26% (28)	21% (15)	0.47
Other	10% (11)	9% (6)	0.71
**Mortality**			
30-day	57% (61)	69% (48)	0.12
90-day	72% (77)	80% (56)	0.23
180-day	77% (82)	87% (61)	0.08

^a^Empiric therapy. ^b^ benzylpenicillin or phenoxymethyl penicillin

*COPD* chronic obstructive pulmonary disease, *ICU* intensive care unit, *IQR* interquartile range

### Mortality

**Fig. 1:**
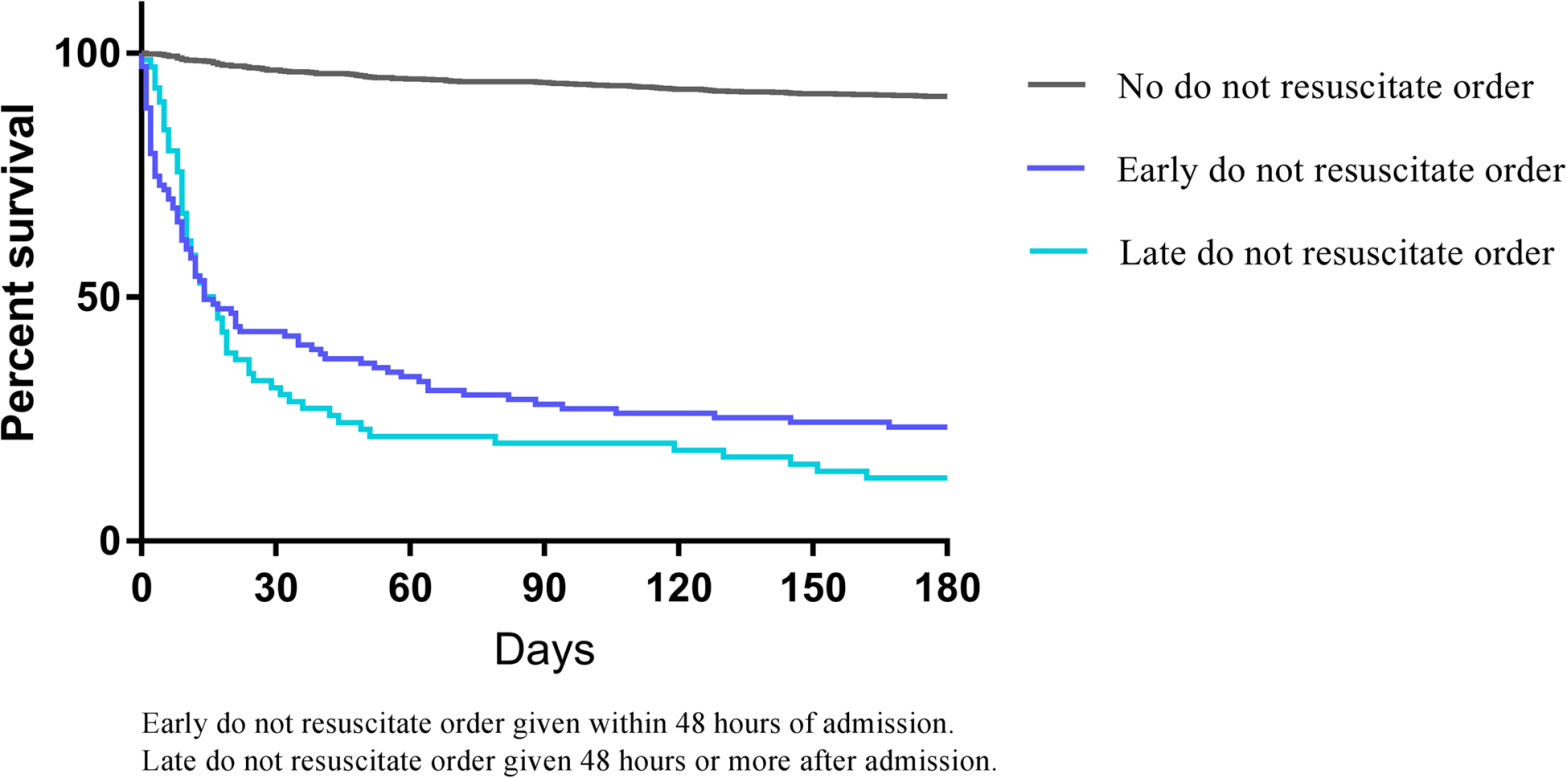
180 days survival according to don not resuscitate order

Patients with a DNR order had higher short- and long-term mortality than patients without a DNR order (30-day mortality: 62% vs. 4% and 180-day mortality: 81% vs. 9%, *p* < 0.001), Fig. 1.

In the cox proportional hazard model, adjusted for age, comorbidities, CURB-65 score, nursing home residency and ICU admission, a DNR order remained significantly associated to 30-day mortality. The interaction between the CURB-65-score and DNR order was significant (*p* = 0.02). The association between a DNR order and 30-day mortality was strongest in patients with low CURB-65 score hazard ratio (HR) 32.4 (95% CI 8.5–123), HR 26.6 (95% CI 12.5–56.7) and HR 9.4 (95% CI 4.7–19) for CURB-65 score 0–1, 2 and 3–5, respectively. Patients with an early DNR order had lower 30- and 180-day mortality than patients with a late DNR order (30-day: 57% vs. 69%, *p* = 0.12; 180-day: 77% vs 87%, yet the difference was not statistically significant (*P* = 0.08). Time from admission to death was median 12 days (IQR: 8–24) in patients with a late DNR order compared with median 10 days (IQR; 2–32) for patients with an early DNR order (*p* = 0.05). In patients with a late DNR order, the order was documented in the record after a median of six days (IQR: 4–13) and the median time from receiving a DNR order to death was 4 days (IQR: 1–9). As a sensitivity analysis we tested the association between an early DNR order and mortality by applying the cox proportional hazard model while ignoring the late-DNR orders which resulted in a HR 3.64 (95% CI 2.48–5.36).

### Risk factors for 30-days mortality in patients without a DNR order

Patients without a DNR order had a 30-day mortality rate of 4%. In the unadjusted cox-regression analysis age, nursing home residency, CURB-65 score and number of comorbidities were all significantly associated with 30-day mortality in patients without a DNR order. In the adjusted analysis only nursing home residency and CURB-65-score 3–5 remained significantly associated with 30-day mortality, HR 6.0 (95% CI: 2.6–13.9, *p* < 0.001) and HR 3.6 (95% CI: 1.1–11.0, *p* = 0.03), respectively (Table 3).

**Table 3:** Risk factors associated with 30-day mortality in patients with no DNR order

	Crude		Adjusted	
	HR (CI)	*P*-Value	HR (CI)	*P*-Value
**Age**	1.5 (1.2–1.9)	0.001	1.0 (0.97–1.04)	0.9
**Nursing home residency**	8.9 (4.6–17.0)	< 0.001	6.0 (2.6–13.9)	< 0.001
**CURB-65 score**				
0–1 (ref)	–	–	–	–
2	3.9 (1.6–9.98)	0.004	2.8 (0.99–8.02)	0.05
3–5	7.3 (2.8–18.9)	< 0.001	3.6 (1.1–11.0)	0.03
**Number of comorbidities**				
None (ref)	–	–	–	–
One	2.9 (1.2–7.1)	0.02	1.5 (0.6–4.1)	0.35
More than one	3.6 (1.5–8.9)	0.006	2.0 (0.7–5.2)	0.18

Number of cases used in the adjusted analysis: 981. HR: hazard ratio. CI: confidence interval

## Discussion

In this cohort of patients hospitalized with CAP, 13% had received a DNR order during hospital admission. More than half received a DNR order during the first two days and again more than half of these died within 30 days. DNR orders were, independent of known risk factors, associated with increased mortality. Conversely, the mortality of patients without DNR orders was markedly lower. Here nursing home residency and severe CAP were significantly associated with an increased risk of mortality.

Surprisingly, only few other studies have described the occurrence of DNR orders in patients with CAP. Similar to our findings, in a US study of 90,644 pneumonia cases from 303 hospitals in California the median DNR-rate was 15.8% [18]. Another US study of 1339 hospitalised CAP patients found that 22% had a DNR order [16]. Further, a Japanese study of 641 CAP patients 65 years of age or older found a DNR-rate of 29% in line with findings from a prospective cohort study of 1093 elderly patients hospitalized with CAP from the Netherlands reporting a DNR-rate of 27,1% [17, 19]. The differences can be due to a larger burden of comorbidities and higher age. Yet, cultural and legislative differences probably also play an important role, which underlines the importance of studying DNR orders in this common group of patients in different settings [21].

As previous researchers [16, 17, 22], we found that almost three quarters of deaths during 30 days were among patients with a DNR order. Even after adjustment for clinical risk factors, patients with a DNR order had an increased risk of both short- and long-term mortality. Walkey et al. [18] also report this association after adjustment for demographics, comorbidities and acute organ failure. This indicates that DNR orders are an important factor to consider when assessing mortality in CAP.

While DNR orders evidently are associated with mortality, some findings warrant further exploration. The association between DNR orders and mortality was strongest in patients with a low CURB-65 score. One explanation for this finding could be that the treating physicians included in their assessment of the patients factors not accounted for in the CURB-65 score such as comorbid conditions and frailty.

Marrie et al. [16] hypothesized that late DNR orders represent a lack of response to treatment whereas they assumed that early DNR orders rather reflect comorbidities and the general health status of a patient. Accordingly, we found that patients with early DNR orders were older and more often nursing home residents. In addition, patients with late DNR orders in our study were more likely to be transferred to the ICU and treated with mechanical ventilation. We did not find a significant difference in the number of comorbidities in patients with late and early DNR orders, albeit there was a tendency towards a higher burden in patients with early DNR orders. Thus, our results largely support the findings by Merrie et al. Since a late DNR order could in some cases represent treatment failure it is possible that including late DNR orders would lead to an overestimation of the association between a DNR order and mortality. Therefore, we performed a sensitivity analysis where patients with a late DNR order were grouped with patients with no DNR order. In the sensitivity analysis an early DNR order remained significantly associated with a higher mortality risk underlining that a DNR order is associated with a higher mortality risk.

An independent association between DNR orders and mortality has previously been demonstrated in other areas of medicine, including patients with septic shock and patients with acute surgical disorders [1, 2, 23]. This leaves the question whether patients with a DNR order died because they received less aggressive and/or inferior treatment or that a DNR order rather mirrors the natural history of CAP in fragile patients. In septic patients with DNR orders, Sakari et al. [2] recorded fewer invasive procedures indicating less aggressive treatment. Further, previous studies investigating physicians’ interpretation of DNR orders have shown that they are misinterpreted to mean that essential steps of treatment such as contacting a doctor upon deterioration, fluid resuscitation and other supportive treatment, may be omitted [24–27]. Furthermore, a DNR order has previously been identified as a negative predictor for ICU admission [28].

In our study patients with CAP with a DNR order did not receive inferior antibiotic treatment or fewer ICU admissions which was also the conclusion from the study by Mulder et al., who concluded that treatment restrictions were not associated with empirical antibiotic treatment in patients with CAP [19]. Thus, we cannot conclude, based on our study, that the excess mortality of patients with a DNR order was caused by inferior treatment. In principle, we cannot exclude that all the patients in our cohort died of a cardiac arrest and thus indirectly of a DNR. However, we find this scenario unlikely. Instead we assume that a DNR order often is a proxy of advanced underlying disease and/or frailty that is not in captured by other established risk factors for mortality.

In a post-hoc analysis, we investigated whether risk factors for mortality in patients with no DNR order differed from those generally found among patients with CAP. We found nursing home residency and disease severity to be risk factors among this group, which is previously reported as important risk factors [3, 12].

The retrospective design is the major limitation of our study because we had to rely on existing data in the patient records. On the other hand, we included all patients with CAP in an entire geographic region thus reducing the risk of selection bias. Obviously, the inclusion of patients with the entire prognostic spectrum is important in a study like ours and we are confident that it reflects “real life” without focus on study patients or adherence to specific treatment regimens. Confounding by indication is a concern because physicians likely are more prone to consider a DNR order in severely ill patients potentially leading to an overestimation of the association between DNR orders and mortality. We could not determine in the patient records whether *not registering a DNR order* was an active choice or merely lack of consideration and we do not know the reason for issuing a DNR order. In addition, we lack data on other treatment limitations such as refraining from intubation and/or admission to the ICU, e.g. some admissions to the ICU could have been for monitoring of inotropic treatment, but not for intubation and cardiopulmonary resuscitation. Whether to consider ICU admission as a confounder may be controversial as some patients may have had an order of no ICU admission and in these cases, it may be considered an intermediate instead. However, as nearly one third of the patients with a DNR order received treatment in the ICU we chose to include it as a confounder. As we lack data on the severity off the individual comorbidities, we have chosen to include them in our model as a grouped variable. There is a risk off residual confounding with this approach, which could have caused us to overestimate the effect of a DNR order.

## Conclusion

In conclusion, a DNR order is an independent risk factor for mortality in patients hospitalized with CAP. Thus, a DNR order is a potential confounder to consider when reporting risk factors for mortality in CAP that has not been taken into account in previous studies. Based on our findings we encourage researchers to account for DNR orders when reporting CAP associated mortality.

## Data Availability

The datasets used and analyzed during the current study area available from the corresponding author in reasonable request.

## Abbreviations

CAP: Community-acquired pneumonia
DNR: Do-not-resuscitate
HR: Hazard ratio
ICU: Intensive care unit
IQR: Interquartile range
LRTI: Lower respiratory tract infection
